# Antibiotic Impregnated Cement Dowel in a Tibial Stump to Salvage Infected Below-Knee Amputation in a Diabetic

**DOI:** 10.5435/JAAOSGlobal-D-18-00042

**Published:** 2019-10-15

**Authors:** Sahil Prabhnoor Sidhu, Neil Jeffrey White

## Abstract

**Case:**

We describe the use of local antibiotics to salvage an infected below-knee amputation in a diabetic patient and prevent the need to convert to the above-knee level. After débridement, an antibiotic tibial “dowel” was fashioned and inserted into the intramedullary canal before closure. The wound was fully healed after four months, and the patient remained satisfied at 15 months.

**Conclusion:**

This case report offers an adjunct to the conventional management of diabetic stump infections. The addition of an antibiotic spacer could offer an alternative to a higher level of amputation and improve the survival rates of below-knee amputation in this difficult population.

Lower extremity amputation is a notable issue for diabetic patients, with diabetic infections being one of the most common indications for lower limb amputation. Two common levels of lower limb amputation are below-knee amputations (BKAs) and above-knee amputations (AKAs), which differ greatly in functional outcomes and quality of life. AKAs place the patient at an increased risk of not being able to use a prosthesis or to ambulate^1^ and are associated with a 50% increase in energy expenditure when compared with BKAs.^2^ Furthermore, AKA decreases the chance of a patient maintaining independent living status^1^ and is associated with increased mortality. Thirty-day mortality rates range from 11% to 18% for AKA versus 4% to 9% for BKA.^3^ Long-term survival rates demonstrate a similar trend, with 1-year survival rates ranging from 50% to 60% for AKA and 65% to 80% for BKA.^3^ Thus, a BKA level should be salvaged whenever possible, such as in the case of a stump infection or wound breakdown.

Management strategies for a diabetic stump infection include the use of broad-spectrum antibiotics with directed therapy after cultures, irrigation and débridement, and conversion to a more proximal amputation if this fails. We present the successful use of a definitive antibiotic spacer to salvage a BKA after a multiorganism stump infection in a diabetic patient. To our knowledge, use of an antibiotic spacer in the setting of an infected diabetic BKA has not been published to date.

## Case Report

A 65-year-old man with a history of poorly controlled type II diabetes mellitus presented with an infected BKA stump. The patient had had multiple previous operations to the affected leg over the span of 5 years, beginning with two partial toe amputations and three débridementsfor infection. He underwent a transmetatarsal amputation, which failed because of wound breakdown and infection. Thus, he underwent a BKA. The wound appeared to be healing 2 weeks postoperatively, and alternating sutures were removed but began to deteriorate shortly after. At 1 month postoperatively, it appeared erythematous, was infected at the skin edge, and had a foul smell. The patient was not septic.

Surgical irrigation and débridement was pursued with 2 g of cefazolin IV administered preoperatively. The wound was opened and dissected down to the level of the bone. There was a slight foul smell and erythematous wound edges. Deep wound swabs were taken, and tissue specimens were sent for culture and sensitivity. We débrided until we were satisfied with healthy-appearing, bleeding tissue. The osteotomy site on the tibia was cleaned using a rongeur. Nine liters of saline with bacitracin was run through the wound. Twelve antibiotic-eluting cement beads were then prepared using 40 g of DePuy cement, 2.4 g of tobramycin, and 2 g of vancomycin. These were placed on a PDS suture, knotted, and placed in the tibia and throughout the wound. The wound was then loosely closed with large PDS mattress sutures and dressed. Two grams of IV cefazolin was administered q8h postoperatively. Ciprofloxacin 500 mg PO BID was also started and discontinued 2 days postoperatively.

On post-op day 2, preliminary deep cultures returned positive for *Staphylococcus aureus* and two unidentified gram-positive organisms. The tissue appeared viable with reasonable vascular supply. Thus, we decided to proceed with revision closure using an antibiotic spacer in an attempt to salvage the stump.

**Figure 1:**
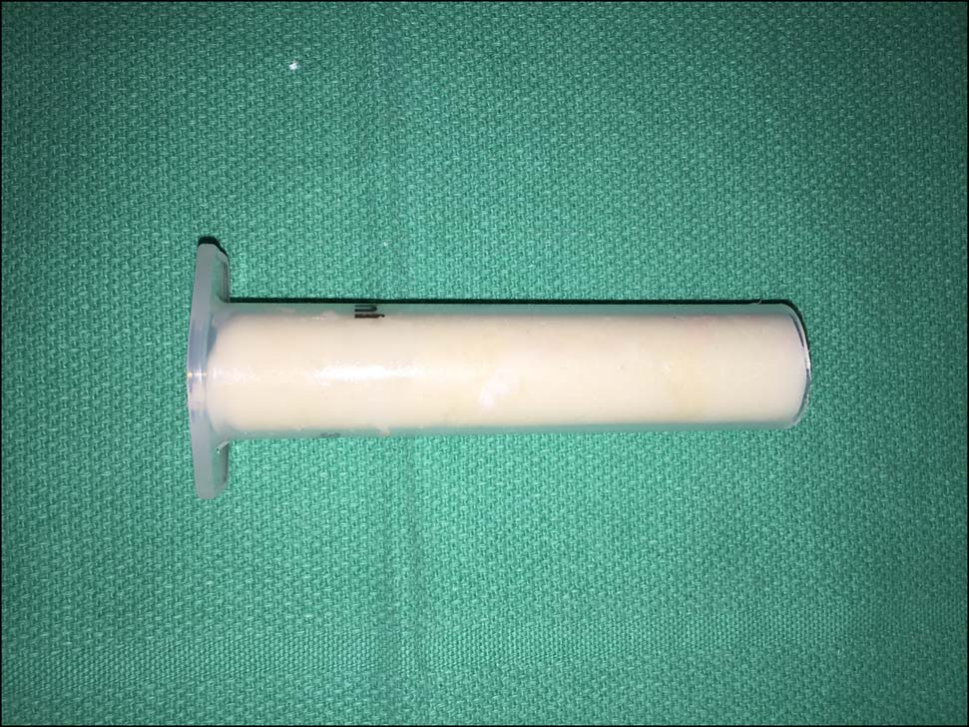
Photograph showing the preparation of a tibial dowel using 2 g of vancomycin and 2.4 g of tobramycin mixed into 1 package of medium viscosity cement. A 20-mL syringe was pretreated with sterile mineral oil to allow for easy removal of the dowel.

**Figure 2:**
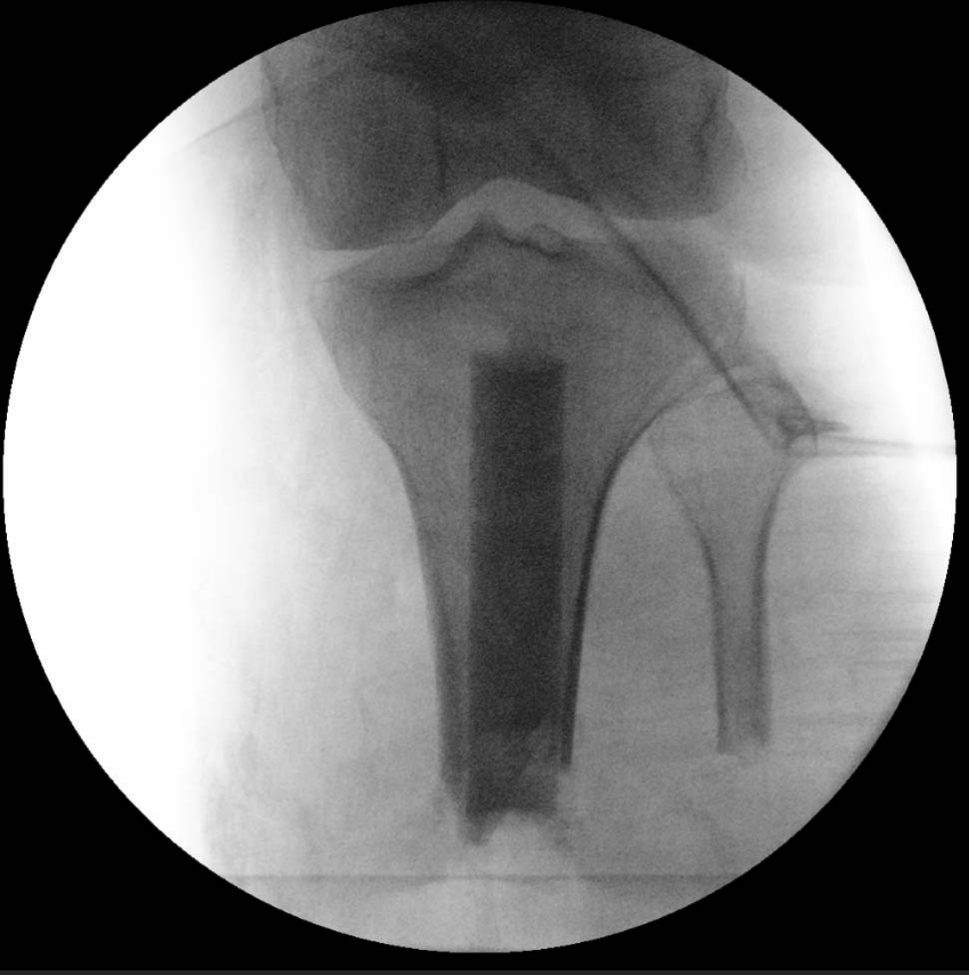
An intraoperative AP radiograph of the left knee showing placement of the antibiotic dowel in the intramedullary canal of the tibia.

Again, 2 g of IV cefazolin was administered preoperatively. We began with a circumferential superficial to deep débridement of all tissue layers. Swabs were sent. A rasp and a rongeur were used to débride the tibia and the fibula. Synthes hand reamers up to 19 mm were used to ream in the proximal tibia within a few millimeters of the subchondral bone under fluoroscopic guidance. The antibiotic spacer was then fashioned using 2 g of vancomycin and 2.4 g of tobramycin (chosen for broad-spectrum coverage) mixed into 1 package of medium viscosity cement. This mixture was used to fill a 20-mL syringe (pretreated with sterile mineral oil to allow for easy removal), allowed to dry, and removed from the syringe (Figure 1). This 18-mm-diameter “dowel” was then placed inside the reamed tibia (Figure 2). The wound was felt to be ready for closure at this time based on the appearance of the tissue and was closed using a spaced combination of #2 Prolene and 3-0 nylon mattress sutures. An incisional VAC dressing was also applied.

Final cultures from the first débridement returned positive for *Staphylococcus aureus* (sensitive to cefazolin, clindamycin, erythromycin, cloxacillin, and sulfamethoxazole/trimethoprim), coagulase-negative *Staphylococcus* (sensitive to clindamycin, erythromycin, sulfamethoxazole/trimethoprim, and vancomycin), and *Corynebacterium jeikeium* (sensitive to vancomycin and gentamycin).

Our infectious diseases colleagues were consulted for antibiotic management. One gram of vancomycin was administered intravenously q12h postoperatively. This was increased to 1.5 g q12h the next day and continued using a pump after discharge. The dose was decreased to 1.25 g q12h at the 2-week follow-up. IV Cefazolin was discontinued 3 days postoperatively. Deep cultures taken during the second procedure produced no growth. The VAC dressing was removed 5 days postoperatively, during which the incision line appeared clean and dry.

At 1 month postoperatively, the incision was still slightly open at the medial aspect with minimal drainage and no evidence of infection. A single-use PICO negative pressure dressing was applied to the wound for 7 days. Vancomycin was decreased to 1 g q12h.

A week later, this dressing was removed and the stump had improved. There were two 5 mm open areas at the center and the lateral edge. All but three sutures were removed.

At 2 months postoperatively, the wound was very small and scabbed over with no evidence of infection. Vancomycin was discontinued, and oral sulfamethoxazole/trimethoprim 800 mg/160 mg was started BID for the first 2 weeks and daily for the next 2 weeks.

At the 3-month follow-up, the wound appeared almost closed with only small scabbing in the center of the suture line. Antibiotics were stopped.

At 14 weeks postoperatively, the patient's course was complicated by a TIA requiring a 2-day admission. He also underwent carotid endarterectomy. Fortunately, he recovered and the wound appeared closed and healthy 4 months after the initial procedure. He was discharged from wound care.

**Figure 3:**
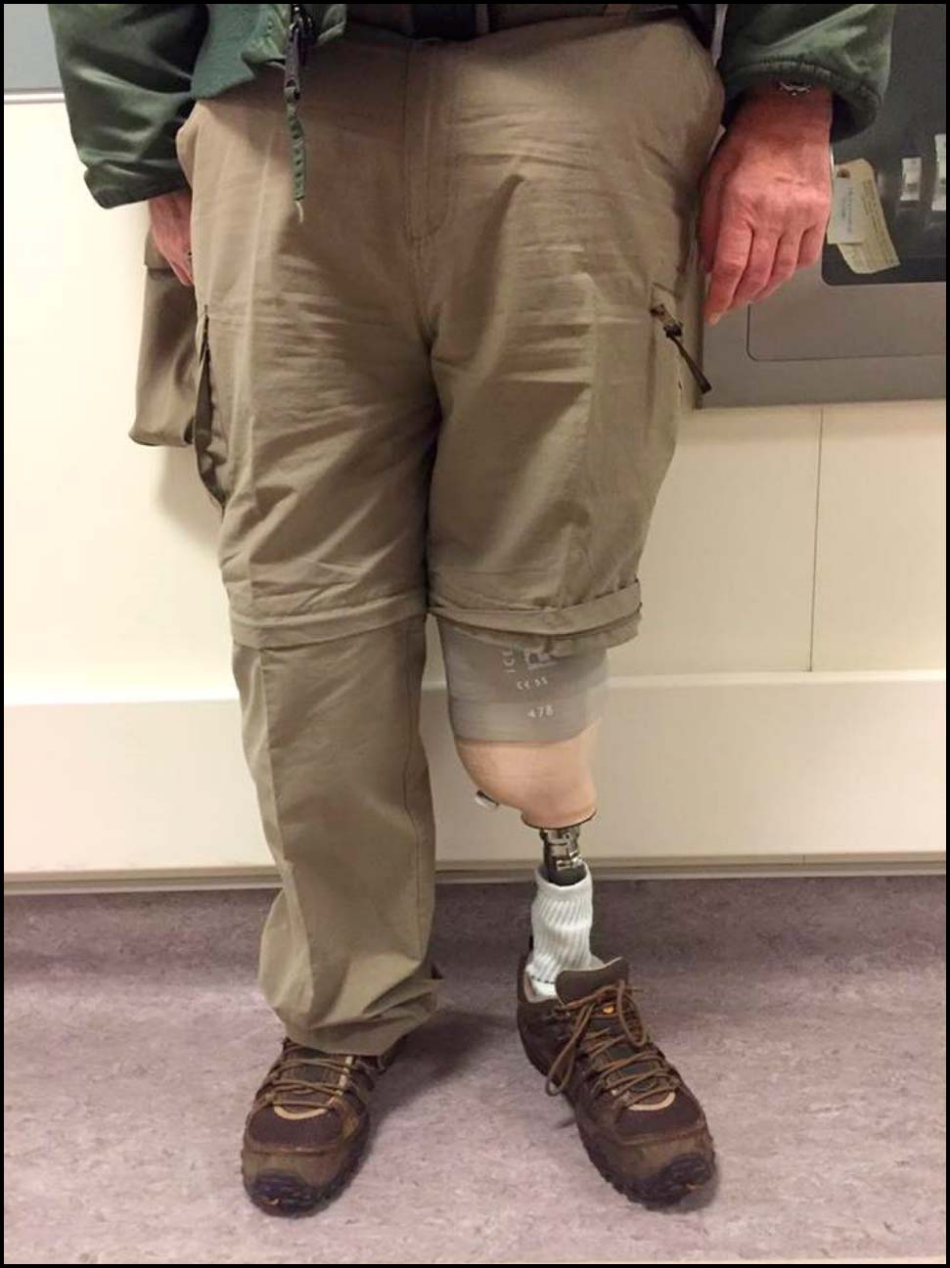
Photograph of a patient who is seen wearing a below-knee prosthesis 8 months after insertion of the antibiotic dowel.

The patient was seen again 8 months postoperatively. The wound was fully healed. He had been fitted with a below-knee prosthesis and was able to walk multiple blocks without difficulty (Figure 3). At 15 months, he scored above average in the physical and mental component scales of the SF-36 survey (52.70 and 58.47, respectively) and the ambulation and well-being components of the Prosthesis Evaluation Questionnaire (92.25 and 95.50, respectively).

## Discussion

Although the use of local antibiotics in the setting of infected BKA has not been published, several studies have demonstrated the efficacy of local antibiotics in other settings. For example, the addition of antibiotics to cement has been shown to decrease infection rates in joint arthroplasty. Similarly, placing antibiotic-loaded PMMA beads into an open fracture site has been shown to reduce acute infection rates in Gustilo type IIIB and type IIIC fractures and the rate of chronic osteomyelitis in type II and type IIIB fractures.^4^

Our rationale for using a definitive spacer revolved around the potential for prolonged local antibiotic exposure. Masri et al^5^ reported elution of vancomycin and tobramycin from cement spacers at levels above the breakpoint sensitivity limit for sensitive organisms at a mean of 118 days when high doses of antibiotic were used. Similarly, Fink et al^6^ found vancomycin, gentamycin, and clindamycin to be eluted in concentrations higher than their MIC from cement spacers 6 weeks after implantation. It has also been shown that antibiotics released from cement can result in local levels that are markedly higher than those reached with parenteral therapy.^7^

The current practice for managing the breakdown of a BKA in a diabetic patient is to initiate the use of broad-spectrum antibiotics and to surgically irrigate and débride the wound. Cultures are used to direct antibiotic therapy. This report offers an adjunct to this current strategy. We believe the addition of a local antibiotic “dowel” could potentially reduce the need to convert infected BKA stumps to the undesirable AKA level. The technique of making a “dowel” inside of a 20-mL syringe is both easy and reproducible. The intramedullary canal of the tibia offers an excellent space to insert local antibiotics without jeopardizing the soft-tissue envelope.

Although more studies are required, this adjunct appears to be relatively safe. Many cases of retained antibiotic spacers have resulted in satisfactory patient outcomes, such as in the case of periprosthetic joint infections^8^ or postarthroscopy shoulder destructive osteomyelitis.^9^ Bone resorption is another concern associated with retained spacers and has been reported in the setting of infected hip replacements; however, this may not be the case in long bones because of the spacer being immobile.^10^ We did not notice any notable resorption.

Currently, this dowel may be used in the setting of known stump infection for both diabetic and nondiabetic patients. We also see a potential role in high-risk patients undergoing BKA with the presence of foot infections or with marginal soft envelopes. This adjunctive intervention appears to offer little downside, removes cancellous bones which can be osteomyelitic in this setting, and fills the dead space. Although more studies are required, this technique may be a consideration in marginal or infected BKA stumps.
